# Share2Quit: Web-Based Peer-Driven Referrals for Smoking Cessation

**DOI:** 10.2196/resprot.2786

**Published:** 2013-09-24

**Authors:** Rajani S Sadasivam, Erik M Volz, Rebecca L Kinney, Sowmya R Rao, Thomas K Houston

**Affiliations:** ^1^Division of Health Informatics & Implementation ScienceQuantitative Health SciencesThe University of Massachusetts Medical SchoolWorcester, MAUnited States; ^2^VA eHealth Quality Enhancement Research InitiativeBedford VAMCBedford, MAUnited States; ^3^Department of EpidemiologyUnviersity of MichiganAnn Arbor, MIUnited States; ^4^Division of Biostatistics & Health Services ResearchQuantitative Health SciencesThe University of Massachusetts Medical SchoolWorcester, MAUnited States

**Keywords:** Web-assisted tobacco interventions, recruitment, peer-driven chain referrals, respondent-driven sampling

## Abstract

**Background:**

Smoking is the number one preventable cause of death in the United States. Effective Web-assisted tobacco interventions are often underutilized and require new and innovative engagement approaches. Web-based peer-driven chain referrals successfully used outside health care have the potential for increasing the reach of Internet interventions.

**Objective:**

The objective of our study was to describe the protocol for the development and testing of proactive Web-based chain-referral tools for increasing the access to Decide2Quit.org, a Web-assisted tobacco intervention system.

**Methods:**

We will build and refine proactive chain-referral tools, including email and Facebook referrals. In addition, we will implement respondent-driven sampling (RDS), a controlled chain-referral sampling technique designed to remove inherent biases in chain referrals and obtain a representative sample. We will begin our chain referrals with an initial recruitment of former and current smokers as seeds (initial participants) who will be trained to refer current smokers from their social network using the developed tools. In turn, these newly referred smokers will also be provided the tools to refer other smokers from their social networks. We will model predictors of referral success using sample weights from the RDS to estimate the success of the system in the targeted population.

**Results:**

This protocol describes the evaluation of proactive Web-based chain-referral tools, which can be used in tobacco interventions to increase the access to hard-to-reach populations, for promoting smoking cessation.

**Conclusions:**

Share2Quit represents an innovative advancement by capitalizing on naturally occurring technology trends to recruit smokers to Web-assisted tobacco interventions.

## Introduction

Smoking is the number one preventable cause of premature death in the United States [1-5]. Although cessation programs have been successfully implemented, the rates of cessation are lower than desired [6]. Effective and easily disseminated interventions such as Web-based smoking cessation websites [7-12,4,13-14] can reach a greater number of smokers [15]. However, these interventions are underutilized [16-18].

Chain-referral methods have rapidly become the methods of choice for recruiting hard-to-reach subjects from their social networks [19-20] and have been used as channels for delivery of a peer-driven intervention [21]. Natural helpers or “Peer Navigators (PNs)” from the community can be trained to effectively deliver health information while increasing access to interventions [22-23]. These “grassroots” and participatory chain referrals unfold in line with the social network dynamics. Hence, PNs facilitate access to high-risk groups, in which individuals are often “like themselves”, within relatively short periods of time. Person-to-person spread of cessation has been a vital factor in the population-level decline in smoking in the recent decades [24]. Furthermore, the decision to stop smoking is not solely an individual one but a reflection of the choices made by groups of people connected to each other [24].

Outside health care, Web-based chain referrals have become quite common to recruit users. Consider the example of the Obama campaign’s use of Facebook to reach unlisted young voters through their friend networks [25] or the example of using peer referrals to recruit more than 75 million users to play a Facebook game called Farmville. However, Web-based chain referrals as a means to recruit patients to online health interventions has not been extensively studied thus far.

Because proactive referrals are more successful than passive referrals [26-29], in our trial, Share2Quit: Web-Based Peer-Driven Referrals for Smoking Cessation, we will build and test a suite of proactive Web-based tools such as email and Facebook referral functions to recruit smokers to a Web-based tobacco intervention system (Decide2Quit.org). We will use respondent-driven sampling (RDS), a controlled chain-referral sampling technique designed to remove inherent biases in chain referrals and obtain a representative sample [30,20]. We hypothesized that smokers with a high social connectivity will have higher numbers of successful recruits. In addition, we hypothesized that the referral success will be higher for women than men. In this paper, we describe our development and research protocol.

## Methods

### Overview

Our goal in the Share2Quit trial is to use chain referrals to recruit smokers to Decide2Quit.org, an evidence-based Web-assisted tobacco intervention system. Decide2Quit.org includes multiple tobacco cessation functions, including tailored patient messaging, secure messaging with tobacco treatment specialists, interactive patient education, and smoking cessation planning [31-33]. On the basis of the projected costs for each sample unit, which depend on the number of seeds we recruit and the number who might complete the follow-up questionnaire, we project a sample size of up to 1200 under the constraint of our budget. The protocol below describes our approach of tool development, and our plan to implement the RDS chain referrals. The Share2Quit study has been approved by the University of Massachusetts Medical School Institutional Review Board.

### Respondent-Driven Sampling

#### Rationale

Chain-referral methods yield a convenience sample that is not necessarily a representative sample of the population of interest. RDS provides a method of quantifying and adjusting for biased samples [30,20]. Using RDS sample weights we may be able to say, for example, whether a certain demographic or risk group is underrepresented in our sample. In addition, RDS methods provide a means of ensuring that our sample is not overly correlated with the initial sample “seeds”, which are non-randomly selected and are likely not representative of the overall population.

Similar to other chain referrals, RDS begins with identifying the initial participants (initial PNs), known as “seeds”, who have a particular characteristic of interest. The initial seeds then recruit individuals from their current social or risk-behavior network for participation in the study. Successive sets of respondents then recruit individuals from their social network for participation. RDS implements behavioral compliance through a group-mediated control triggered by secondary incentives [20]. Incentives are of two types: primary incentives that are given directly to an individual for completing a task, and secondary incentives that are given to a peer to elicit participation from another peer. Secondary incentives are more powerful because peers have better access to participants and can overcome barriers, influence participation, and more effectively monitor participants [20].

RDS has several key requirements, including [30] (1) the population being recruited must be socially networked and (2) starting with the PN seeds, each participant is allowed to recruit no more than a pre-specified number of recruits (a recruitment quota). Quotas are an important control for PNs with larger networks to prevent over-recruiting from among their peers, and thereby creating biased samples with shorter chains [34-35,20]. Online, the quotas will also reduce the potential of recruiting solely for incentives because the total incentive is limited.

We will implement multiple strategies as described below to keep the number of seeds and the number of recruitments per sample unit low, and thus reduce the correlation between sample units. A recent critique [36] of RDS showed that high levels of assortativity (like-with-like preferential attachment) within the social network can drastically increase the variance of RDS estimators. Assortativity is the propensity with which the nodes of similar connectivity are connected to each other [36-37]. However, the efficiency of the sampling method can be considerably improved using few simple modifications. We propose the following two innovations to RDS.

#### Innovation 1: Adaptive Sampling Strategy to Decrease Intra-Sample Correlation

Intra-sample correlation describes how strongly units in the same group describe each other that is, it measures the relatedness of two individuals in the chain [38]. A previous study showed that the covariance between sample units in a chain-referral design is a decreasing function of distance between the sample units in the recruitment chain, where n is the sample size, n_S_ is the sample size for units with state *S* (eg, a given race or age profile), d_uv_ is the distance between units u and v in the recruitment chain, and σ is a matrix of Markovian transition probabilities and the estimated proportion of the population with state *S* [39].

A straightforward way to decrease the intra-sample correlations and thereby increase the precision of samples obtained using RDS is to modify the sampling process in such a way that this average distance is increased. We have devised an adaptive sampling algorithm to address this issue. The algorithm operates by finding an optimal recruitment quota, which changes as the sample is collected. The quota is selected in a way that maximizes the probability that the sample will be collected at the desired rate (recruitments per unit time) while minimizing the probability of the recruitment chain from dying out.

RDS samples have significant uncertainty in the timing and logistics of sample collection because the referral process depends on the behavior of the study population that is not known ahead of time. For example, the speed of sample collection depends on the number of recruitments made per respondent (Share2Quit PN) and the average time between initial recruitment of the PN and subsequent referral. This problem also affects the decision of the number of seeds to be use to initialize the sample.

To address the problems of sample design with respect to seed selection and choosing an appropriate recruitment quota, Dr Volz developed the respondent-driven sampling simulator (RDSS) [40], which will allow us to simulate thousands of recruitment trees given the underlying parameters such as the recruitment quota and number of seeds. The main output of these simulations is the length of time required to achieve the desired sample size, as well as the probability of sample failure (the probability that the recruitment tree does not achieve the required sample size because of non-response). This information will allow us to determine the optimal number of seeds and recruitment quota.

Additional sample design considerations must be made because both referrals and the study surveys are expected to take place online. We have recently gained valuable insights into Internet-based RDS by conducting a study [41] of 3448 young adults (ages 18-24) across the United States designed to assess Internet use, drug use, and sexual risk behaviors. Several findings from this study can be applied to Share2Quit. To prevent fraudulent recruitment, such as when an individual adopts multiple personas with the objective of interviewing multiple times, it is important to actively observe the chain-referral process for warning signs, such as multiple recruitments from related IP blocks and email addresses. Online recruitment is generally much faster than standard coupon-based recruitment. Certain demographics, especially the highly educated white males and Asians, have been observed to recruit at a much higher rate than the other sociodemographic groups. To prevent this trend from skewing the composition of the sample toward these demographics, it is essential to control the interval between interviews of the recruiter and those that are nominated by the recruiter. This is accomplished by tuning the time between the interview and when our system sends out the invitation to the nominated individuals.

#### Innovation 2: Improved Estimation of Sample Weight

Methods for estimation of sample weight may utilize sociometric degree as well as the assortative mixing patterns of respondents. Several competing estimates were compared in a recent study, which showed that one of the most simple estimators is also relatively stable and accurate. Therefore, in this study, we intend to use variations of the RDS2 estimator [42]. We propose to use additional sociometric information not ordinarily collected during RDS studies. We used the counting procedure of McCarty et al [43] and assessed the sociometric degree in categories such as age, race, gender, and the type of relationship. This approach not only yields accurate estimates of the total degree but also provides information about assortative mixing patterns in the underlying social network. Information of the assortativity patterns enables us to devise accurate estimates of sample inclusion probabilities; for example, if most recruitments are made by men, we would adjust upwards the estimated inclusion probability for someone who reports knowing many men. We call the estimator that uses this auxiliary estimator RDS2+[39]. These new methods have not been implemented in an empirical setting thus far, and Share2Quit provides an appropriate test-bed.

### Preliminary Concept of the Intervention

Initially, we will describe our preliminary concept of the intervention and then describe how we will refine it by using user input. The tools that we plan to develop for Share2Quit are as follows:

- Automated recruitment messages: PNs can use a secure email or Facebook form to market the intervention. Once the smoker completes an online consent form, the PNs can refer this individual to the intervention simply by entering his/her email or Facebook ID into a secure form. The referred smoker will then receive a series of 10 automated emails or Facebook messages encouraging him/her to register on the intervention website.
- Feedback reports and persuasive message templates: After making initial referrals, the PNs will be provided feedback reports on the registration status of their recruits. The system will allow the PNs to prompt referred smokers to register by using specific messaging templates (a menu of motivational and informational messages that the PNs may choose to modify). Templates will be created as communication facilitators, so PNs navigators do not have to create messages but can just “pick and send”
- Social networking widgets: Share2Quit will have a menu of social networking widgets (eg, quit smoking progress counter and link to a “chemicals in smoking game”). These widgets allow PNs to share information on Decide2Quit.org with their social network.

**Figure 1:**
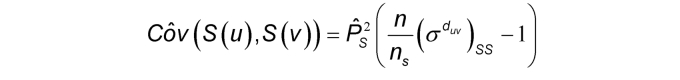
Equation for covariance between sample units in a chain-referral design.

The goal of these components is to recruit and engage new smokers to the intervention site and, subsequently, recruit the same group of smokers to chain-refer within their social networks. Share2Quit will begin with the recruitment of an initial set of seed PNs. The flow of the referral “lifecycle” of a single PN through the Share2Quit intervention is shown in Figure 1. To initiate the intervention, a PN (P1) will use the Share2Quit tools to engage and recruit a current smoker from their social network to register on the intervention website. The current smokers (Wave1-1, Wave1-2, etc) will then register on intervention site and will begin to use the Decide2Quit.org system. As part of the system, after their first visit to the website, these new smokers will be recruited to engage as PNs, so that they may also refer current smokers in their social network (W2-1, etc). We will adhere to the best practices of RDS and maintain a low quota, but the actual number will be based on our formative work and the adaptive sampling strategy described above.

**Figure 2:**
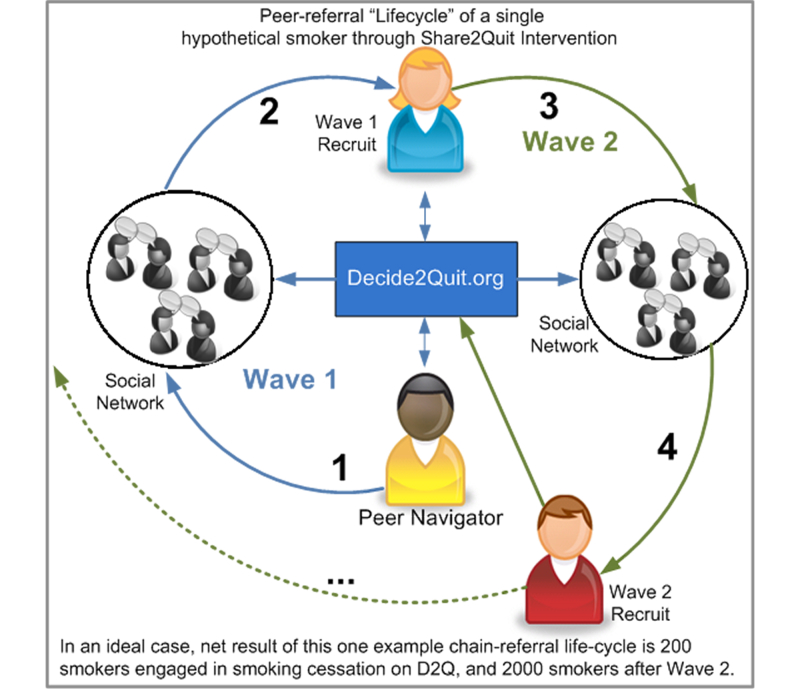
Peer-referral “Lifecycle” of a single hypothetical smoker through Share2Quit Intervention: 1: Seeds or peer navigator consents to be in the study and refers smokers from his network using S2Q tools. The system contacts the referred smokers and prompts them to register. 2: The referred smokers register on the system and consents to become a peer navigator (Wave 1 recruit). 3: The Wave 1 recruit then refers smokers from their social network and similarly the system prompts these smokers to register. 4: The referred smokers then registers on the system (Wave 2 recruit). 5: The chains progress until the target sample size is reached.

### Designing the RDS Chains Using RDS Simulations

We will perform RDS simulations to provide information about the design of our chains. RDSS requires the following data to simulate the RDS chains: (1) time required for recruitment and (2) number of expected recruits per seed (ie, how many social contacts the respondent thought would be open to chain-referral recruitment). Thus, our first step will include an online survey of smokers (n=50). We will recruit these smokers through Google advertisements. We will assess the number of smokers an online smoker is connected with, the strength of these social ties, and the time to recruit. In addition, we will assess prior referral behavior and willingness to refer to a Web-assisted tobacco intervention system (see Multimedia Appendix 1 for our draft survey instrument).

### Integrating Share2Quit Tools With Our Previous Work (Decide2Quit.org) Using an Agile Methodology

The Web-based Decide2Quit.org was programmed using Microsoft’s ASP.NET version 3.5 (Microsoft Corporation, Redmond, WA, USA) and C# technology. We used Microsoft SQL Server version 2000 as the database. Decide2Quit.org was programmed using a modular architecture, which makes it easier to add Share2Quit functionalities. We will develop Share2Quit functions using an Agile methodology that is characterized by its phased and collaborative nature [44]. Unlike traditional approaches, the Agile methodology recommends forming an overall strategy and then collaboratively developing a system in phases. This approach is especially advantageous in a research setting because developers can easily adapt to changing requirements. Share2Quit functions will be developed in compliance with Health Insurance Portability and Accountability Act (HIPAA) standards using Secure Socket Layer technology.

### Usability Testing of Chain-Referral Tools

#### Overview

Current and ex-smokers (5-10) will be recruited from central Massachusetts to perform individual in-depth interviews alpha testing the potential human-computer interface. We will use the "Think Aloud" protocols described by Kushniruk [45,46] as follows: while the participants are reviewing the content and interacting with the program, they will be asked to vocalize their thoughts, feelings, and opinions. Think Aloud allows you to understand how the user approaches the interface and what considerations they have in mind when utilizing the interface. Usability sessions will be conducted by the UMass Division of Health Informatics mobile usability lab and Morae usability software. Morae [47] has successfully been utilized in testing Web-based software [48,49] and enables live remote observation of the subject being tested (eg, recording of clicks, keystrokes, and other events). In addition, Morae allows for annotation of the usability sessions by the observer. Prompts will be used to elicit the response on any item for which the user has not provided feedback (see Multimedia Appendix 2 for sample usability prompts). We will exclude these pilot test participants from the main study.

#### Interview Analysis

Interviews will be transcribed and anonymized. There are several approaches to evaluating qualitative data, including thematic analysis, narrative summary, and grounded theory [50]. Because our primary goal is to understand the process of using Share2Quit, we will use a combination of thematic and narrative analyses. The transcribed interviews will be reviewed by two independent reviewers to develop preliminary themes. To develop themes, we will use an open-coding approach to be maximally inclusive. Each open-ended question in the interview guide will be assessed separately, and then the reviewer will generate larger summary themes for the overall interview. The themes will then be reviewed with the larger investigator group to resolve disagreements. From the themes, we will create summary tables of key points. We will complete this method twice while collecting interviews. Thus, we will assess for theme saturation and further revise our data collection methods to focus on details of interest for the second wave of interviews. Narrative summary is best used when qualitative data follow a logical order [51]. On the basis of the interview transcriptions and example Share2Quit tools provided to smokers, we will develop Share2Quit workflow process diagrams to be used in system development and education of PNs.

### Intervention

#### Overview

The intervention will begin with the recruitment of seeds. Current and former smokers above the age of 21 will be eligible to participate as seeds. We will recruit seeds using multiple approaches. Initially, we will recruit from the current cohort of smokers already registered in Decide2Quit.org. Between 2010 and 2012, we recruited 1777 smokers in our current R01 (NIH 5R01CA129091-04). We will email these smokers offering them the opportunity to participate in Share2Quit and refer smokers. In addition, we will recruit seeds through Google and Facebook advertisements. Once the seed consents using an online form, he or she will be provided access for 30 days to refer the smokers. We will begin with a recruitment quota of 3 as recommended in the RDS best practices, but we may increase the quota on the basis of RDSS simulations as described above on the actual referral data. Once these seeds refer smokers, newly registered smokers will also be offered the opportunity to recruit additional smokers. Seeds and PNs will have to complete an informed consent form before being provided with the referral tools. Each smoker will be provided a monetary incentive for recruiting smokers.

#### Data Collection and Analyses

We will collect data at multiple points in time, including at registration with our system, at agreement to be a PN, at referral, and at follow-up. We will use our existing Web analytics tracking program to monitor use of the system. We will use this tracking data and determine our primary outcome variables of referral success. These include the number of successful new recruits per recruiter and the length of subsequent referral chains. All PNs and recruits will be connected through a unique identifier. Follow-up will be conducted after seeds and PNs have 30 days of access to the tools. We plan to perform 3 analyses: an evaluation of the impact of each component tool of Share2Quit, a comparison of the characteristics of chain-referred smokers to those recruited through physician practices, and identification of predictors of successful recruitment chains (adjusted using RDS sample weights). In addition to the new smoker registration, we have expanded data collection to include the pre-referral (PN survey), during referral, and follow-up time periods (Table 1 and Multimedia Appendix 3 [41,52-55]).

**Table 1:** Key data elements.^a^

Data elements	Parameters tested
New smoker registration	Demographics (age, gender, ethnicity, education, marital status)
	Smoking-related comorbidities
	Allow smoking at home
	Number of cigarettes smoked per day
	Quit in last 12 months
	Want to stop smoking
Peer-navigator survey	Number of estimated smokers in network (family, friends, and acquaintances)
	Previous website referrals
	Number of subjects willing to be referred
	Syme/Berkman Social Network Index [52,53]
Share2Quit referral form	Demographics of referred smoker (age, sex, race)
	Nature of relationship with referrer
	Length of relationship
	Number of interactions
	Tools used for interactions
PN follow-up survey	Number of subjects attempted to refer
	Sociometrics of refused (friend, family, or acquaintance)
	Influence on PN smoking
	Satisfaction with Share2Quit tools
Decide2Quit.org Web tracking (all users)	Number of logons, pages visited, time on page, etc
	Number of referrals
	Number of new registrations

^a^All instruments linked through unique RDS ID codes to connect recruitment waves

#### Comparing Samples

Our new smoker registration survey includes demographic questions, tobacco use behaviors, and previous participation in Web-based smoking cessation sites. We will compare the RDS sample characteristics with those of our current samples obtained from physicians and dentists by conducting a bivariate analysis.

#### Tool Success

In this study, we will allow PNs to select the tools they use. We will track the use of these tools online. Then, we will compare the impact of each tool and tool combination on measures of referral success

#### Primary Analyses

RDS-sample weighted network analysis (predictors of successful referrals): As discussed, we will use RDS to generate a sample of chain-referred smokers. In previous Web-based RDS analyses, the investigators noted that some sociometric “stars” who become hubs are highly successful at referring. We propose analyses to understand which characteristics of those referred can be used to predict registration, and which characteristics of the PNs can be used to predict successful chain referrals.

We propose to evaluate age, sex, ethnicity, and measures of social connectedness. We hypothesize that PNs who report high social connectivity will have higher numbers of successful recruits. In addition, we hypothesize that referral success will be higher for women than men. Inferences about the social network will be drawn from data collected during recruitment, and this data will be used to create sample weights. All analyses will use these sample weights generated from the RDS to make inferences for the population of interest. Our primary analysis will assess the association of social connectedness and a measure of referral success. Our outcome variable will be the number of referrals by a PN. The independent variable will be the Social Network Index from the adapted Syme/Berkman social network scale (I=lowest score, IV=highest score) [53]. This scale comprises 4 components (marital status, contacts with friends and family, membership in groups, and group associations), and we will use them for our study. We will first compare bivariate associations, and then perform multivariable modeling using ordinal logistics adjusted for variation in tool use and other predictors significant in bivariate association or those that need to be included because of our conceptual rationale.

### Power Calculations for Comparing the Association of Referral Success and Social Connectedness

Our main hypothesis is that people who are highly socially connected (as measured by the social network scale [52,53]) will have a higher proportion of referrals than those not very socially connected. Although we expect a sample up to 1200, power calculations for RDS samples must acknowledge the association between recruits and are adjusted for a design factor (up to 2) [36]. Thus, for power calculations, we have reduced the real sample size of 1200 to an adjusted, effective sample size of 600 to be conservative (Table 2). While the recent work by Goel et al [36] suggests that a more appropriate design effect for power calculations would be 5-10, we believe on the basis of stochastic simulation that the modifications to the sample design (such as the adaptive recruitment quota) will shrink the design effect to 2-3. As discussed, we will use an adaptive sampling strategy, varying timing and quotas dynamically under the guidance of Dr Volz. To simplify power calculation, we assume that each PN will be able to recruit up to 2-3 new smokers because of our quota. Although each PN has a truncated range of recruitment (eg, 0-3 recruits out of 3 possible), when averaged over groups of PNs, we can approximate a proportion. We based the power calculation to detect a significant difference in the proportions of successful referrals in two groups on the basis of dichotomization of the social network scale. We assume that a certain number of the 600 referees will be highly socially connected and will have a certain proportion of successful referrals. We show detectable differences in Table 2. Sample size of 300 in each group achieves 80% power to detect a difference between the group proportions of -0.06 if group one has 10% of successful referrals (successful referrals in group two will be 4% or 0.04) and can still detect a 11% difference if group one has 50% successful referrals (successful referrals in second group will be 39% or 0.39). The test statistic used is the two-sided Z test with pooled variance and a targeted alpha level of 0.05. Calculations were made using PASS [56]. Because of the small number of seeds and a restricted range, the proportion (or count) may not represent a continuous variable. In addition to standard approaches, we will use quantile regression if required depending on the distribution of the outcome. Quantile regression is a different method of approaching central tendencies and dispersion that can be used for categorical outcomes.

**Table 2:** Power calculations for comparing the association of referral success and social connectedness.

Number of highly socially connected peer navigators (N_1_)	Number of peer navigators not highly socially connected (N_2_)	Proportion of successful referrals among N_1_	Absolute detectable difference
300	300	0.1	0.06
300	300	0.5	0.11
200	400	0.1	0.06
200	400	0.5	0.12
100	500	0.1	0.07
100	500	0.5	0.15

## Results

This protocol describes the evaluation of proactive Web-based chain-referral tools, which can be used in tobacco interventions to increase the access to hard-to-reach populations, for promoting smoking cessation.

## Discussion

Our overall goal is to increase access to a smoking cessation intervention by recruiting PNs (former and current smokers) who will be trained to refer current smokers from their social network. Previous studies and our preliminary data suggest multiple barriers in the reach of smoking cessation interventions, including those delivered online. Thus, new approaches are required to increase the reach of these interventions [57].

Peer navigation leverages current online trends because social network tools are increasingly popular. For example, Facebook has over 99 million users [58]. On an average, users spend 55 minutes daily on Facebook, maintaining 130 “friends”, writing 25 content-related critiques, and rating via the “like” button 9 times per month [59]. Facebook users are more likely to trust peer referrals to products [60-62]. Moreover, social network referral tools such as the “like” and “become a fan” mechanisms, are pivotal in attracting new users. A recent survey showed that 41% of respondents claim that they joined a Facebook fan page to communicate to their friends what products they support [60-62]. These findings are not limited to one demographic group. In addition to Whites (31%), Hispanics (50%), Asians (46%), and African Americans (44%) consider social networks a useful tool for researching new products [61].

To our knowledge, Share2Quit will be the first study to test online social networks and chain referrals to proactively recruit smokers to a Web-based smoking cessation intervention. Thus, Share2Quit represents an innovative advancement by capitalizing on a combination of naturally occurring technology trends to recruit smokers to our Decide2Quit.org Internet-based cessation intervention. Drawing from our previous Web-based smoking intervention work, Share2Quit will test a suite of tools designed to allow PNs to market and prompt current smokers within their social network to participate in a cessation site. Share2Quit PNs will be selected from a pool of smokers currently participating in Decide2Quit.org. Given that the PNs will be connected to the smoker(s), recruitment messages are more likely to be personally relevant and more effective than conventional advertisements. Share2Quit will implement best practices of RDS and the recent innovations of WebRDS, including the use of multiple strategies to keep the recruitment quotas low, adaptive sampling to decrease intra-sample correlation, and collection of additional sociometric information to enhance sample weights. Thus, our approach is uniquely suited to recruit smokers that may otherwise be considered hard-to-reach [19]. We will track the referrals through unique identifiers to better facilitate an analysis of the social networks.

Our study has some limitations. Like most online light-touch interventions, the data we are collecting on the PNs is mostly through self-report, and we cannot confirm the veracity of these data. Because our study is a highly innovative, high-risk study, considerable uncertainty is present in our sample size calculation, and we may have overestimated this number.

## Multimedia Appendix 1

Share2Quit interview.

## Multimedia Appendix 2

Share2Quit interview with usability prompts.

## Multimedia Appendix 3

Key data elements.

## Abbreviations

HIPAA: Health Insurance Portability and Accountability Act
PN: peer navigator
RDS: respondent-driven sampling
