# Supplementary material for: Share2Quit: Web-Based Peer-Driven Referrals for Smoking Cessation
Source: JMIR Res Protoc. 2013 Sep 24;2(2):e37. doi: 10.2196/resprot.2786 (PMC3786127; doi:10.2196/resprot.2786)
Supplement: Supplementary file 1 [file resprot_v2i2e37_app1.pdf]

Appendix 1: Share2Quit Interview

Share2Quit In-depth Interview

[DRAFT]

(This is a draft interview guide with the questions we would ask in the formative interview participants. The questions will be refined in the first few months of the project)

Thank you for taking the time to participate in our Share2Quit study. Your thoughts and suggestions to the following questions are appreciated and will assist us in building the Share2Quit intervention site.

1. How often do you ever refer your friends and family to website?

1. Never
2. Occasionally
3. Frequently

2. If yes (q1), have you ever referred your friends and family to a health website?

1. Never
2. Occasionally
3. Frequently

3. Have you ever been referred to website by your friends and family?

1. Never
2. Occasionally
3. Frequently

4. If yes (q3), did you visit the website?

1. No
2. Yes

5. If yes (q3), have you ever been referred to a health website by your friends and family?

4. Never

1. Occasionally

2. Frequently

6. If yes (q5), did you visit the health website?

1. No

2. Yes

7. Please estimate how many of your friends and family are smokers?

    1. 0
    2. 1-5
    3. 6-10
    4. 10-20
    5. >20

8. Thinking about the individuals that you know who smoke cigarettes, how many are:

1. Immediate family members, *number*_____

2. Extended family members or relatives, *number*_____

3. Close friends, who you feel at ease with and discuss private matters, *number*_____

4. Friends, who you feel at ease with but who you DO NOT discuss private matters, *number*____

5. Acquaintances, persons who you may say “hello” to but know little about, *number*____

6. Co-workers, *number*____

9. Would you be willing to refer your friends and family who are trying to quit smoking to an online smoking cessation website?

    1. Yes
    2. No
    3. Maybe

10. How many of your friends and family who are smokers do you think would be open to being referred?

    *number*____

11. How long of a time-frame would you estimate you need to refer your friends and family?

    1. Couple of days
    2. 1 week
    3. 1- 2 weeks
    4. 2 – 4 weeks
    5. > 4 weeks

12. When you interact with these individuals, who smoke cigarettes, which mode of communication are you most likely to utilize?

** Drop down field for each response?

a. *Face-to-face*

b. *Phone*

c. *Email*

d. *Social networking (Facebook, etc.)*

e. *Other web option, specify* ________

1. **Immediate family** members, _____

2. **Extended family** members or relatives, _____

3. **Close friends**, who you feel at ease with and discuss private matters, _____

4. **Friends**, who you feel at ease with but who you DO NOT discuss private matters, ____

5. **Acquaintances**, persons who you may say "hello" to but know little about, ____

6. **Co-workers**, ____

Referring someone from your social network would require filling out the online referral form (5-10 minutes) and encouraging your referral to participate by registering at the online website.

Question 13 will be randomized (some participants will be asked to respond to 13a and others, 13b).

13a. What is least amount of monetary incentive that would motivate you to refer your friends or family who are smokers to an online smoking cessation website.

____

OR

13b. What is least amount of monetary incentive that would motivate you to refer your friends or family who are smokers to an online someone cessation website.

1. 0-5
2. 5-10
3. 10-15
4. 15-20
5. 20-25
6. Other ------------------------------

Demographics:

13. Which category best describes your current age?

1. 19-24 years
2. 25-29 years
3. 30-39 years
4. 40-49 years
5. 50-59 years
6. 60 +

14. What is your gender?

1. Male
2. Female

15. Which category best describes your ethnicity?

1. Not Hispanic or Latino
2. Hispanic or Latino

16. Which category best describes your race?

1. White
2. Black or African American
3. Asian
4. Native Hawaiian or other Pacific Islander
5. American Indian/Alaska Native
6. Other, specify:___________________

17. Which category best describes the highest year of schooling that you have completed?

1. Less than high school
2. High school graduate
3. Some college
4. College graduate
5. Post baccalaureate

If you are interested in providing us further input on the specific tools we are developing, please check the box below. Our research assistants will contact you to setup a convenient time.

Yes, I am interested in receiving more information, a good time to contact me would be:_____________ am/pm

Thank you for completing the survey. To receive your gift card, please enter your email address and phone number below. Expect gift card delivery to your email (remember to check your junk mail) 1-2 days after the completion of this survey.
Email address:
Phone number

Thank you.
