# Supplementary material for: Share2Quit: Web-Based Peer-Driven Referrals for Smoking Cessation
Source: JMIR Res Protoc. 2013 Sep 24;2(2):e37. doi: 10.2196/resprot.2786 (PMC3786127; doi:10.2196/resprot.2786)
Supplement: Supplementary file 2 [file resprot_v2i2e37_app2.pdf]

Appendix B: Sample Usability Prompts

Share2Quit In-depth Interview

DECIDE2Quit Usability

1. In the Share2Quit study, we are developing a system in which smokers and/or ex-smokers can refer others smokers to a smoking cessation website. This website will have a host of tools and resources that may be utilized to aid in smoking cessation efforts. What are your thoughts on such a resource? What type of tools would you like to see in such a resource?

2. See screenshot/see website for our initial concept of the referral tools including an email referral and Face book App

1. Tell me your thoughts on these two functions?

____

- What did you like about these functions?
- What would you change about the functions?

2. What do you think about us providing templates that you can edit in your message to your friends and family?

- Would you use them as is?
- Would you edit them?

____

2. How would you use these functions to refer your friend and family who are smokers?

____

3. Once you refer, would you prompt your friends to register on the system?

____

4. Did these functions make you think about quitting?

____

<u>Demographics</u>:

3. Which category best describes your current age?

1. 19-24 years
2. 25-29 years
3. 30-39 years
4. 40-49 years
5. 50-59 years
6. 60 +

4. What is your gender?

1. Male
2. Female

5. Which category best describes your ethnicity?

1. Not Hispanic or Latino
2. Hispanic or Latino

6. Which category best describes your race?

1. White
2. Black or African American
3. Asian
4. Native Hawaiian or other Pacific Islander
5. American Indian/Alaska Native
6. Other, specify:___________________

7. Which category best describes the highest year of schooling that you have completed?

1. Less than high school
2. High school graduate
3. Some college
4. College graduate

8. Would you like to participate in future usability testing like this one.

Email:
Phone Number:

Thank you.
