# Supplementary material for: Share2Quit: Web-Based Peer-Driven Referrals for Smoking Cessation
Source: JMIR Res Protoc. 2013 Sep 24;2(2):e37. doi: 10.2196/resprot.2786 (PMC3786127; doi:10.2196/resprot.2786)
Supplement: Supplementary file 3 [file resprot_v2i2e37_app3.pdf]

Appendix 3: Key Data Elements

New Smoker Registration Form

1. What is your age?

1 <19 **END SURVEY**
2 19-24
3 25-34
4 35-44
5 45-54
6 55-64
7 65+

2. For which of the following activities do you routinely use the Internet? (check all that apply)

0. Not at all
1 Search for information on topics such as news, health, travel, sports
2 Read information on a website
3 Send or read e-mail
4 Watch videos or listen to audio clips
5 Download files such as computer software, videos, or pictures
6 Use an online social networking site like MySpace or Facebook or blogging
7 Engage in online activities that require more input such as purchasing items, playing games, banking

2. Do you allow smoking in your home?

1 Yes
0. No

4. About how many cigarettes do you smoke per day?_____ (number)

5. Have you ever visited a smoking cessation website?

1. Yes
0. No

6. During the past 12 months, have you stopped smoking for one day or longer because you were trying to quit smoking?

1. Yes
0. No

7. Do you want to stop smoking cigarettes?

2. I do not smoke now

1. Yes
0. No

**Did anyone <u>at the doctor's office that referred you to this website</u> do any of the following:**	**Yes, at my last visit**	**Yes, at another visit**	**No**
8. Ask you if you smoke cigarettes?			
9. Advise you to quit smoking cigarettes?			
10. Refer you to the 1-800-QUIT-NOW tobacco quitline?			
11. Arrange a follow-up visit or call to talk more about your quitting smoking?			
12. Prescribe nicotine patches, gum or lozenges to help you quit smoking cigarettes?			
13. Give you any handouts or pamphlets about tobacco use?			

14. Are you…?

0. Male
1. Female

15. Do you consider yourself to be Hispanic or Latino, that is a person of Mexican, Puerto Rican, Cuban, South or Central American, or other Spanish culture or origin regardless of race?

1. Hispanic or Latino
2. Not Hispanic or Latino
98. Don't Know/Not Sure
99. Refused

16. What RACE do you consider yourself to be? (*Select one or more of the following)*

1. Black or African American
2. White
3. Asian
4. American Indian or Alaska Native
5. Native Hawaiian or Other Pacific Islander
6. Other: (specify)____________________
98. Don't Know/ Not Sure
99. Refused

17. What is the **HIGHEST GRADE OR YEAR OF SCHOOL** you completed?

1. Never attended school or only attended kindergarten
2. Grades 1 through 8 (Elementary)
3. Grades 9 through 11 (Some high school)

4. Grade 12 or GED (High school graduate)
5. College 1 year to 3 years (Some college or technical school)
6. College 4 years or more (College graduate)

98. Don't Know/Not Sure
99. Refused

18. Please provide a name for the Decide2Quit system to call you. This can be your first name or a nickname. We have personalized the system to use your "name" when you log on!

1. Name: ____________________________________.

19. Please provide contact telephone numbers for a study coordinator to call you to complete a follow-up survey. We will need to complete this survey in order to provide the $30.00 gift card as compensation associated with this portion of the study.

1. Primary phone________________________(home or cell)
2. Secondary phone ______________________ (cell or other)

THANK YOU!

PEER-NAVIGATOR SURVEY

Initial Questionnaire Instrument

Thank you for your interest in the Share2Quit Intervention. We would like to understand the number of people you know who smoke cigarettes and how these individuals may fit into specific categories; these people could be friends, acquaintances, family, and coworkers. These questions may sound strange, but they help us to understand the board categories of people within your social network who smoke cigarettes. We appreciate your participation.

1.) How often do refer your friends and family to a website?

0 Never

1 Occasionally

2 Frequently

2.) Have you ever been referred to a website by your family or friends?

0 Never

1 Occasionally

2 Frequently

3.) If yes, have you ever visited a site referred by your family or friends?

0 No

1 Yes

4.) Which best describes your marital status?

0 Married

1 Divorced [GO TO QUESTION #6]

2 Widowed [GO TO QUESTION #6]

3 Separated [GO TO QUESTION #6]

4 Never married [GO TO QUESTION #6]

5 Member of an unmarried couple

5.) Is your spouse/partner a smoker

0 No

1 Yes

6.) Please estimate how many of your family and friends are smokers?_______

7.) Thinking about the individuals who you know that smoke cigarettes, how many are ** Drop down field for each response?

a) **Immediate family** members, _____

b) **Extended family** members or relatives, _____

c) **Close friends**, who you feel at ease with and discuss private matters,

d) **Friends**, who you feel at ease with but who you DO NOT discuss private matters, ____

e) **Acquaintances**, persons who you may say "hello" to but know little about, ____

f) **Co-workers** _____

8.) How many of your friends and family THAT SMOKE, would be open to being referred to Decide2Quit.org? _________

9.) How much time would you estimate that you would need to refer your family and friends to the website? ________

10.) When you interact with your family and friends who smoke cigarettes, which mode of communication are you most likely to utilize?

0 Face-to-face

1 Phone

2 Email

3 Text Message

4 Social Networking

5 Other web option, specify:________________

11.) Do you participate in any groups, such as a social or work group, religious-connected group, self-help group, or charity, public service, or community group?

0 No
1 Yes
9 Unknown

12.) Do you use online social networks to interact with your friends/family (eg: Facebook, Google+, Twitter, MySpace)?

0 No

1 Yes

9 Unknown

13.) Thinking about those who you would refer what would they call you (Nickname)?

_____

Share2Quit Referral Form

Referral #1:

We would like to better understand who you are referring to the Decide2Quit website. Please use the fields below to describe the person you are referring:

How do you address this individual (nickname): ______________________

Their relationship with you: [Drop-down]

a.) Your spouse or partner

b.) Immediate family member (other than your spouse)

c.) Extended family members and/or relatives

d.) A close friend (a person you feel at ease with; can talk to about private matters)

e.) Friend (someone you know personally, like, & trust but whom you do not share private matters)

f.) Acquaintance (someone you may say “hello” to, but know little about)

g.) Co-Worker

h.) Other:__________________________

What is their age: [Drop-down]

a.) 18-24

b.) 25-29

c.) 30-34

d.) 35-39

e.) 40-44

f.) 45-49

g.) 50-54

h.) 55-59

i.) 60-64

j.) 65-69

k.) 70-74

l.) ≥75

What is their sex:

a.) Male

b.) Female

c.)

Please use this email form to message your friend. You can personalize the message below

Please enter their email address:____

Email subject: *Together we can stop smoking: check out this link!*

Email Message:

Dear [name of friend],

Recently, I have been participating in a smoking cessation website called Decide2Quit. This website offers many tools and resources to help smokers in their cessation efforts. One of the benefits of the website is that it allows for those already registered, such as myself, to refer their family and friends to these beneficial and effective cessation resources.

I would like to invite you to the Decide2Quit website. Simply click on the following link to register: www.decide2quit.org. Together, we can support each other in our cessation efforts and finally become tobacco free.

Good luck!

Sincerely,

PEER NAVIGATOR
Follow-up Survey

1. With how many other smokers did you discuss our website, www.decide2quit.org ?
____

2. With those smokers mentioned in Question 1 above, which modes of communication did you use? (please check all that apply)
   1. Face-to-face

2. Telephone
3. Email
4. Facebook or a social networking site
5. Text message

3.For those smokers mentioned in Question 1 above, what was the most common reason a smoker would **refuse** referral to the www.decide2quit.org website?

1. Not ready to quit smoking
2. No computer/internet access
3. Cannot use computer/website (lack of technical abilities)
4. No time
5. Other, please specify:_________________________

4. We asked you to recruit family and/or friends to the www.decide2quit.org website.

Please indicate your agreement with the below statements: (adaptation of STORIES SIS)

Recruiting Smokers to Decide2Quit:

	Strong Agree	Agree	Neutral	Disagree	Strong Disagree
a.) Was beneficial to my own quit smoking efforts.					
b) Motivated me to get support from those around me to quit smoking					
c.) Increased my craving for cigarettes.					
e.) Made me feel like I was being helpful to my family and friends who are smokers					
f.) Was burdensome					

5.) Please indicate your agreement with the below statement:

	Strong Agree	Agree	Neutral	Disagree	Strong Disagree
a. The referral tools provided at the www.decide2quit.org website were helpful in my referral efforts:					
b. The instructions provided at the www.decide2quit.org website were clear and easy to follow:					

6.) We asked you to **recruit at least 3 family and/or friends** to the www.decide2quit.org website.

You referred: XXXX

a) We gave you a time period of 1 month to complete your recruitment.

Please indicate your agreement with this statement:

	Strong Agree	Agree	Neutral	Disagree	Strong Disagree
Yes, I could have recruited more people to the www.decide2quit.org website if I was provided more time					

If Strong Agree or Agree to 6a, how many more people could you have referred, ______________ *(number) (BASED ON ANSWER DISPLAY)*

b.) We provided $15 per recruitment.

Please indicate your agreement with this statement:

	Strong Agree	Agree	Neutral	Disagree	Strong Disagree
Yes, I could have referred more people to the www.decide2quit.org website if the incentives were doubled ($30 per referral)					

If Strong Agree or Agree to 6c, how many more people could you have referred, ______________ *(number) (BASED ON ANSWER DISPLAY)*

7. Please indicate your agreement with this statement.

You are receiving the incentives one-time after a period of one month at the end of the study.

	Strong Agree	Agree	Neutral	Disagree	Strong Disagree
Instead of receiving my incentives at one time, I would have preferred receiving an incentive after each of my referrals registered at www.decide2quit.org					

Thank you for participating in the Decide2Quit study
